# Bilateral Eyelid Edema in a Pediatric Patient With COVID-19: A Case Report and Literature Review

**DOI:** 10.7759/cureus.40427

**Published:** 2023-06-14

**Authors:** Shunsuke Shimazaki, Junichi Sato, Atsuko Niwa

**Affiliations:** 1 Department of Pediatrics, Funabashi Municipal Medical Center, Chiba, JPN

**Keywords:** child and adolescent, ocular symptoms, acute dacryoadenitis, covid-19, eyelid edema

## Abstract

Severe acute respiratory syndrome coronavirus 2 (SARS-CoV-2) is primarily transmitted through the eyes, nose, or mouth. Ophthalmic complications, such as conjunctivitis and dacryoadenitis, have been reported in patients with coronavirus disease 19 (COVID-19). We report the case of an early adolescent girl who presented with bilateral urticarial rashes, eyelid edema, fever, and cough. She was diagnosed with acute dacryoadenitis with SARS-CoV-2 infection confirmed by a nasopharyngeal polymerase chain reaction and clinical investigations. The patient was treated with dexamethasone (3 mg daily) for three days, which resulted in the resolution of fever and urticarial rash, and improvement of eyelid edema. While bilateral upper eyelid edema and acute dacryoadenitis commonly occur in pediatric patients due to Epstein-Barr virus (EBV) infection and Kawasaki disease, they are rarely associated with other diseases. However, ocular symptoms have been reported in 11.4% of patients with COVID-19. In addition, eyelid edema and acute dacryoadenitis have also been reported after COVID-19 messenger RNA (mRNA) vaccination. The underlying mechanisms of these complications are not yet completely understood. Our case highlights the possibility of bilateral eyelid edema in children with COVID-19, which can occur in addition to other viral infections such as EBV.

## Introduction

The coronavirus disease 2019 (COVID-19) pandemic has affected individuals of all ages worldwide. Severe acute respiratory syndrome coronavirus 2 (SARS-CoV-2) infection occurs as a result of direct contact with the eyes, nose, or mouth [1]. In addition to respiratory symptoms, the SARS-CoV-2 infection can cause complications in various organs, including the eyes. Conjunctivitis and dacryoadenitis have been reported as ophthalmic complications of COVID-19. Their underlying mechanism is not yet fully understood, and they are considered rare in pediatric patients.

Bilateral upper eyelid edema and acute dacryoadenitis are rare in pediatric patients but have been reported in association with infectious diseases such as Epstein-Barr virus (EBV) infection [2]. Additionally, EBV symptoms can include clamped nasolacrimal anatomy, and the infection can induce nasal mucosal congestion and acute nasolacrimal duct obstruction.

In this report, we describe a rare case of bilateral eyelid edema in an adolescent patient with COVID-19. Our case highlights the importance of recognizing the potential ocular complications associated with COVID-19 in children and adolescents.

## Case presentation

**Figure 1:**
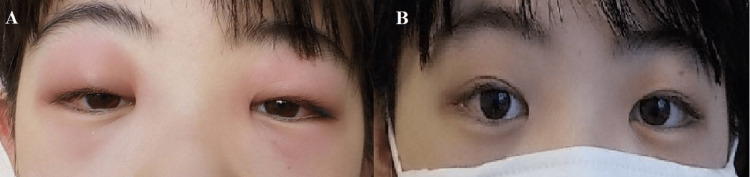
COVID-19-related eyelid edema Photograph of the patient on admission showing a bilateral urticarial rash and swelling around both eyes (A). Photograph of the patient three days later showing improvement of the rash and swelling of the eyes after treatment with dexamethasone (B).

A 12-year-old girl presented to the pediatric department of our hospital with a one-day history of bilateral eye swelling, fever, and cough. She had no history of allergies or other illnesses. Physical examination revealed bilateral urticarial rash and eyelid edema (Figure 1A). Clinical examination revealed no ocular discharge and ocular movements were within the normal range. The patient’s body temperature was 39.5 ℃. Blood tests showed a white blood cell count of 7400/µL (reference range, 3300-8500/µl) and a C-reactive protein (CRP) level of 2.39 mg/dL (reference, <0.3 mg/dL). Hepatic transaminase, creatinine, bilirubin, and lactate dehydrogenase levels were normal. Immunoglobulin (Ig)M and IgG antibody titers against Epstein-Barr virus (EBV) capsid antigen, and IgG antibodies against EBV nuclear antigen, were negative, indicating no prior EBV infection. The patient was diagnosed with SARS-CoV-2 infection, confirmed using nasopharyngeal swab polymerase chain reaction (PCR). In addition to SARS-CoV-2 infection and based on the clinical and laboratory investigations, the patient was diagnosed with acute dacryoadenitis. Eight months ago, she received a second dose of the COVID-19 mRNA vaccine (BNT162b2/Cominaty, Pfizer-BioNTech). Her brother had been diagnosed with SARS-CoV-2 several days ago.

The chest radiograph was normal. The patient was admitted to our hospital for fever and fatigue and was started on antihistaminic drug treatment. A dose of 3 mg dexamethasone daily (in three divided doses) was added to her treatment regimen one day later, as her symptoms did not improve. After three days of treatment, her fever resolved, and the urticarial rash and eyelid edema improved (Figure 1B). Dexamethasone therapy was terminated in three days. The patient was discharged from the hospital. A follow-up visit three weeks later revealed a complete resolution of symptoms with no symptom recurrence since then.

## Discussion

Bilateral upper eyelid edema and acute dacryoadenitis are rare in pediatric patients but have been reported in association with infectious diseases such as EBV infection [2]. EBV symptoms can include clamped nasolacrimal anatomy, and the infection can induce nasal mucosal congestion in addition to the acute obstruction of the nasolacrimal duct. However, in this case, the patient tested negative for EBV antibodies. In addition to infectious diseases, eyelid edema can also be observed in Kawasaki disease. Moreover, Komatsu et al. reported the presence of the purple eyelid sign in children with multisystem inflammatory syndrome (MIS-C) [3]. In the present case, our patient was not diagnosed with MIS-C or Kawasaki disease since she did not report any gastrointestinal symptoms and thus did not meet the diagnostic criteria for complete or incomplete Kawasaki disease [4]. Based on the described evidence, we considered acute dacryoadenitis as the most likely cause of eyelid edema.

Ophthalmic complications, including conjunctivitis and dacryoadenitis, have been reported in adult patients with COVID-19. In a meta-analysis, 11.4% of patients with COVID-19 reported ocular symptoms, including ocular pain, redness, and follicular conjunctivitis [5]. Ophthalmic complications were, in fact, the first manifestations in 2.26% of patients with COVID. Kase et al. reported a case of chronic bilateral dacryoadenitis in a patient with COVID-19, which showed SARS-CoV-2 positive inflammatory cells with glandular damage [6]. Indeed, glandular damage was not observed in the patient with dacryoadenitis and was negative for COVID-19. This suggests that lacrimal gland ducts may be targets for SARS-CoV-2 adhesion. As a result, the retrograde spread of SARS-CoV-2 from the tear film to the lacrimal glands may cause infectious dacryoadenitis. A histopathological examination of the urticarial rash was not conducted in this case, as the patient’s symptoms promptly improved.

Eyelid edema and acute dacryoadenitis have been reported to develop after COVID-19 mRNA vaccination [7]. In fact, antibodies against the spike glycoproteins present in the mRNA vaccine may trigger an acute autoimmune response. In this way, eyelid edema may result from an autoimmune and inflammatory response triggered by the mRNA vaccine. However, we did not consider the vaccine as a causal factor for these conditions, as the patient had received the vaccine more than half a year ago.

 Steroid therapy has been reported to be effective for eyelid edema in those patients with SARS-CoV-2 infection categorized as moderate or high, who require oxygen administration. In our case, dexamethasone therapy was initiated after one day of antihistaminic drug treatment, as the eyelid edema did not improve. Indeed, after three days of treatment, her symptoms improved, and at the three-week follow-up visit, the patient had completely recovered. This case supports the current view that steroid therapy is effective for eyelid edema in patients with SARS-CoV-2 infection [6,7].

## Conclusions

Here, we report a case of bilateral eyelid edema associated with SARS-CoV-2 infection in a pediatric patient. Bilateral eyelid edema is rare in children with COVID-19. However, it should still be considered a possible diagnosis. Furthermore, ophthalmic complications can be the first manifestation of COVID-19, thus a prompt diagnosis and management may prevent serious sequelae.
